# 95. Novel Antifungal Olorofim Use in Intractable CNS and Non-CNS Disseminated Coccidioidomycosis

**DOI:** 10.1093/ofid/ofae631.032

**Published:** 2025-01-29

**Authors:** Fariba Donovan, George R Thompson, Royce H Johnson, Rasha Kuran, Thomas F Patterson, Martin Hoenigl, Joanna M Schaenman, Shmuel Shoham, Steven M Holland, Monica K Sikka, Andrej Spec, Mark Bresnik, John N Galgiani, John H Rex

**Affiliations:** University of Arizona, Tucson, AZ; University of California Davis Medical Center, Sacramento, CA; Kern Medical Center, Bakersfield, California; Kern Medical Center, Bakersfield, California; University of Texas Health San Antonio, San Antonio, TX; Division of Infectious Diseases, Department of Internal Medicine, Medical University of Graz, Graz, Austria, Graz, Steiermark, Austria; University of California Los Angeles, David Geffen School of Medicine, Los Angeles, California; Johns Hopkins University School of Medicine, Baltimore, Maryland; National Institutes of Health, Bethesda, Maryland; Oregon Health and Science University, Portland, Oregon; Washington University in St. Louis, St. Louis, MO; F2G, Ltd., Princeton, New Jersey; University of Arizona, Valley Fever Center for Excellence, Tucson, Arizona; F2G, Limited, WELLESLEY HILLS, MA

## Abstract

**Background:**

Disseminated coccidioidomycosis (DCM) requires long courses of antifungal treatment. Olorofim, a novel antifungal in development, selectively blocks pyrimidine biosynthesis by inhibiting fungal dihydroorotate dehydrogenase (DHODH), and we evaluated its impact on clinical and mycologic response in patients with refractory DCM.

**Figure:**
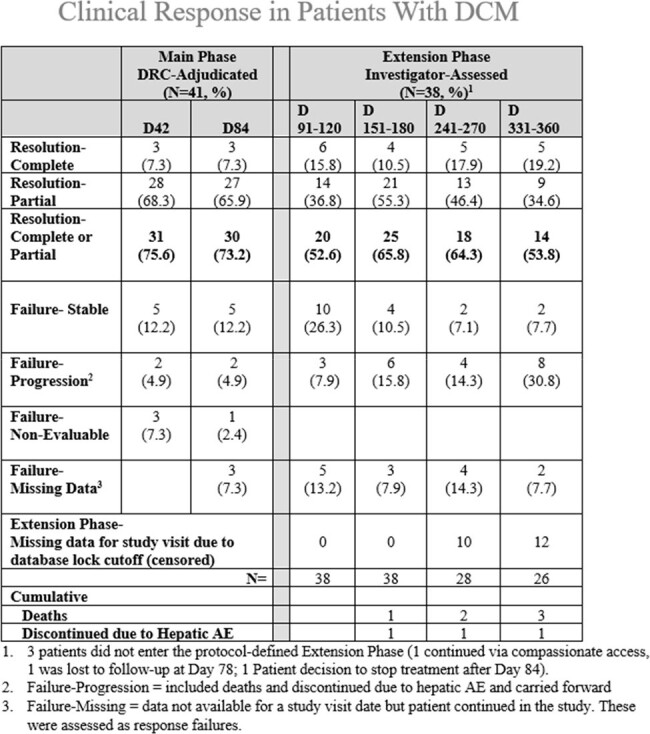

**Methods:**

Adults >18y with limited or no treatment options enrolled in NCT03583164 (Study 32), an open label olorofim Phase 2b study. A Main Phase of 84+6 days was followed by an Extension Phase; we report here outcomes through 1 year.

**Results:**

41 DCM patients enrolled at 10 US sites from 05/2019 to 08/2022. Median age was 47y (range 21-72y). 30 (73.2%) had CNS disease, of whom 13 (43%) had ventriculoperitoneal shunt or Ommaya reservoir. Median time from DCM diagnosis to olorofim initiation was 936d (range 47d to 8y). Median (mean) olorofim treatment was 353d (382d).

Data Review Committee (DRC)-adjudicated EORTC-MSG was Partial Clinical Response (PCR) in 28 (68.3%) at Day 42 and 27 (65.9%) at Day 84. Complete Clinical Response (CCR) was in 3 (7.3%) at both timepoints. Constitutional findings (fever, malaise) often resolved within 4 weeks. Integrated Overall Response (Clinical + Mycologic + Imaging) was 0% at Day 42 & 84 due to slow resolution of serologic findings of DCM, even in patients with CCR. Patients in the Extension Phase had PCR or CCR ranging 52.6% to 65.8% with lower rates due, in part, to missing data at study visit dates being assessed as failures, in addition to the fluctuating clinical status of DCM patients over time.

Hepatic injury, judged at least possibly related to olorofim was 9 (22.0%) in Main Phase, was managed by dose reduction/pause, and led to discontinuation in 1 (2.4%) patient. Non-serious and self-limited GI symptoms were noted in 16 patients (39%) of which 1 (2.4%) was related to olorofim. Three deaths during Extension Phase were not attributed to olorofim.

**Conclusion:**

Olorofim has a positive benefit-risk profile in DCM patients with no or limited treatment options. Favorable Clinical Responses were seen in 73.2% and often sustained through 1 year of treatment. Hepatic injury was usually manageable with monitoring. Future studies should assess earlier use of olorofim in serious DCM.

**Disclosures:**

**Fariba Donovan, MD/PhD**, F2G: Grant/Research Support **George R. Thompson, III, MD**, Astellas: Advisor/Consultant|Cidara: Advisor/Consultant|Cidara: Grant/Research Support|F2G: Advisor/Consultant|F2G: Grant/Research Support|Melinta: Advisor/Consultant|Melinta: Grant/Research Support|Mundipharma: Advisor/Consultant|Mundipharma: Grant/Research Support|Pfizer: Advisor/Consultant **Thomas F. Patterson, MD**, F2G: Grant/Research Support **Martin Hoenigl, MD**, Aicuris: Advisor/Consultant|Astra Zeneca: Honoraria|Gilead: Grant/Research Support|Gilead: Honoraria|IMMY: Grant/Research Support|Melinta: Grant/Research Support|Melinta: Honoraria|MSD: Grant/Research Support|Mundipharma: Grant/Research Support|Mundipharma: Honoraria|Pfizer: Grant/Research Support|Pulmocide: Advisor/Consultant|Pulmocide: Grant/Research Support|Scynexis: Advisor/Consultant|Scynexis: Grant/Research Support|Shionogi: Honoraria **Joanna M. Schaenman, MD, PhD, FAST**, Eurofins Viracor: Honoraria|F2G: Grant/Research Support|MedCure: Advisor/Consultant|Moderna: Clinical trial support to institution|OneLegacy: Advisor/Consultant **Shmuel Shoham, MD**, F2G: Grant/Research Support **Monica K. Sikka, MD**, F2G: Grant/Research Support **Andrej Spec, MD, MSCI**, F2G: Grant/Research Support **Mark Bresnik, MD**, F2G Ltd: Employee **John H. Rex, MD**, F2G: Employee

